# Supplementary material for: Imageological/Structural Study regarding the Improved Pharmacokinetics by 68Ga-Labeled PEGylated PSMA Multimer in Prostate Cancer
Source: Pharmaceuticals (Basel). 2023 Apr 14;16(4):589. doi: 10.3390/ph16040589 (PMC10144514; doi:10.3390/ph16040589)
Supplement: Supplementary file 1 [file pharmaceuticals-16-00589-s001.zip › pharmaceuticals-2290274-supplementary.pdf]

Supporting Information for

Imageological/structural study regarding the improved pharmacokinetics by ^{68}Ga-labeled PEGylated PSMA multimer in Prostate Cancer

Huihui Zhang[1†], Maohua Rao[1†],Huayi Zhao[1], Jianli Ren[3], Meng Zhong[4], Lan Hao[3], Yue Chen[2], Xia Yang [5],Yue Feng [2*], Gengbiao Yuan[1*]

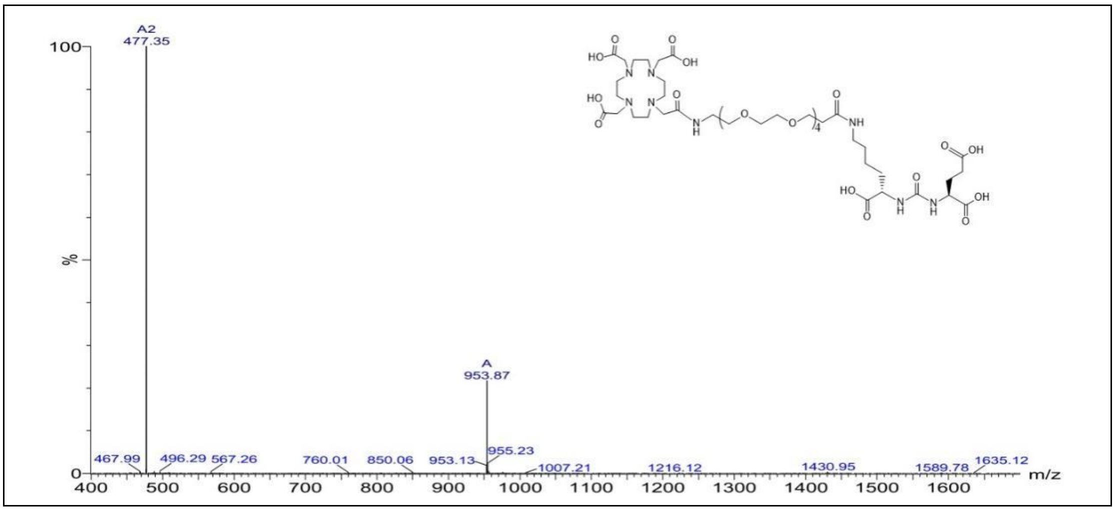

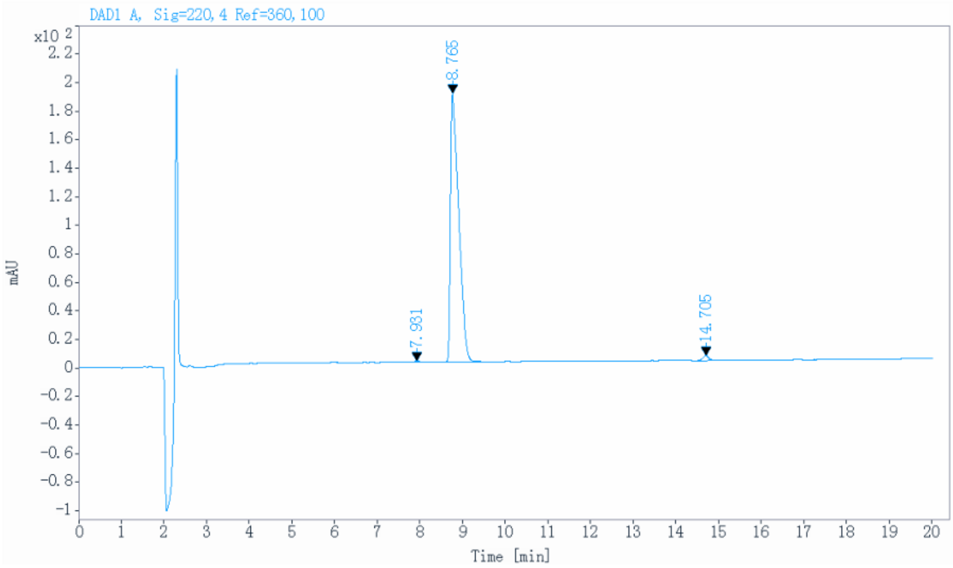

Signal: DAD1 A, Sig=220, 4 Ref=360, 100

RT[min]	Type	Width[min]	Area	Height	Area%	Name
7.931	BB	0.1075	5.2580	0.7350	0.2041	
8.765	BB	0.2113	2536.7080	188.1719	98.4531	
14.705	BB	0.1493	34.5984	3.5006	1.3428	
		Sum	2576.5644			

Figure S1. MS spectroscopy and HPLC of DOTA-(1P-PEG$_4$)

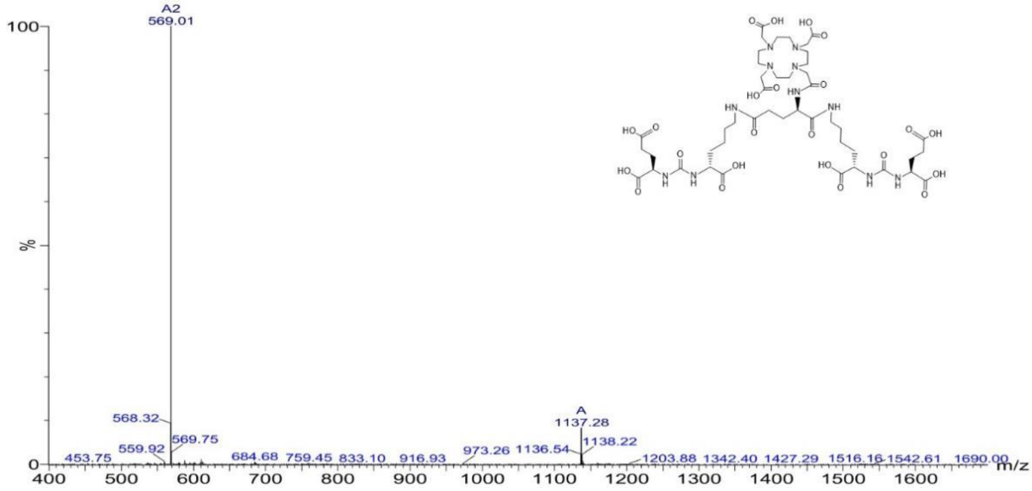

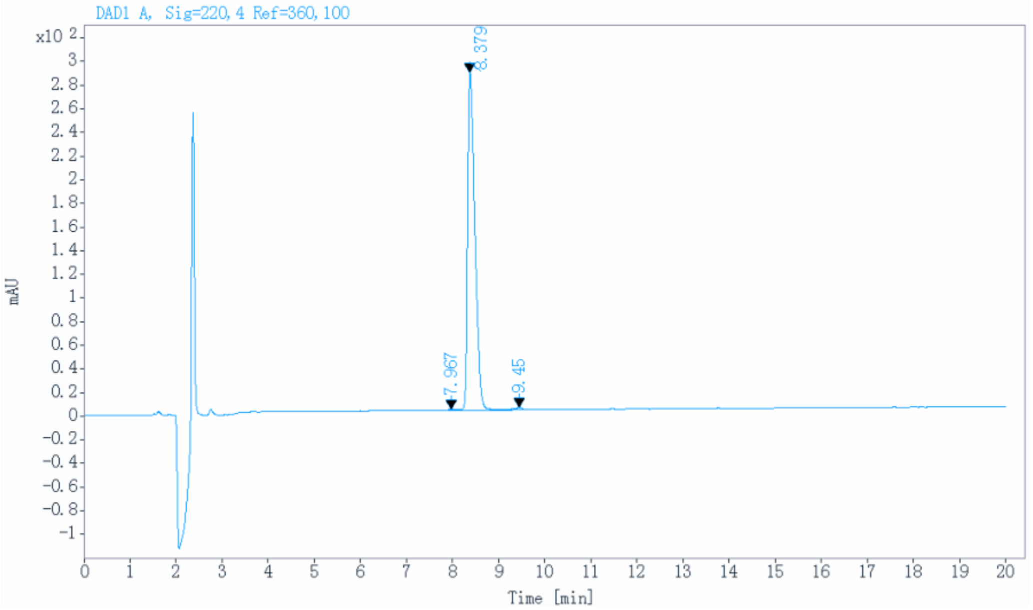

Signal: DAD1 A, Sig=220,4 Ref=360,100

RT[min]	Type	Width[min]	Area	Height	Area%	Name
7.967	BV	0.1163	3.4712	0.4039	0.1110	
8.379	VV	0.1643	3096.5374	285.9850	98.9950	
9.450	VB	0.1789	27.9638	2.1334	0.8940	
		Sum	3127.9724			

Figure S2. MS spectroscopy and HPLC of DOTA-(2P-PEG$_0$)

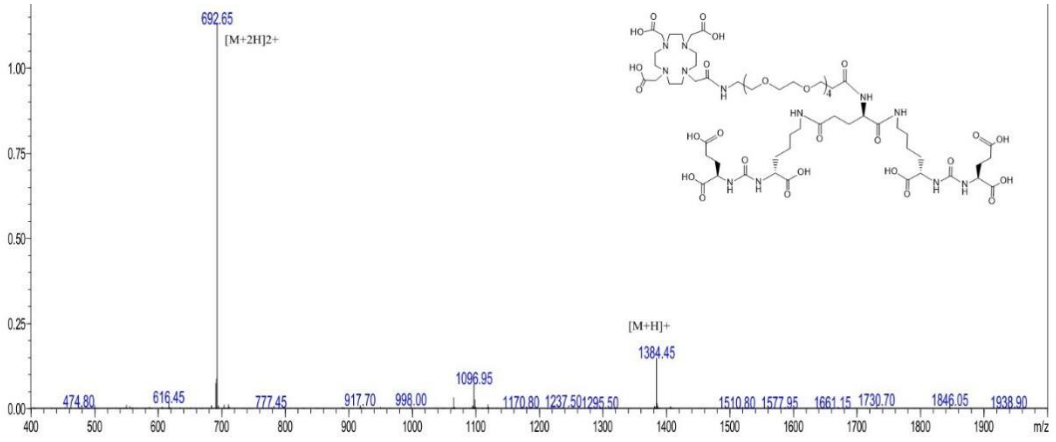

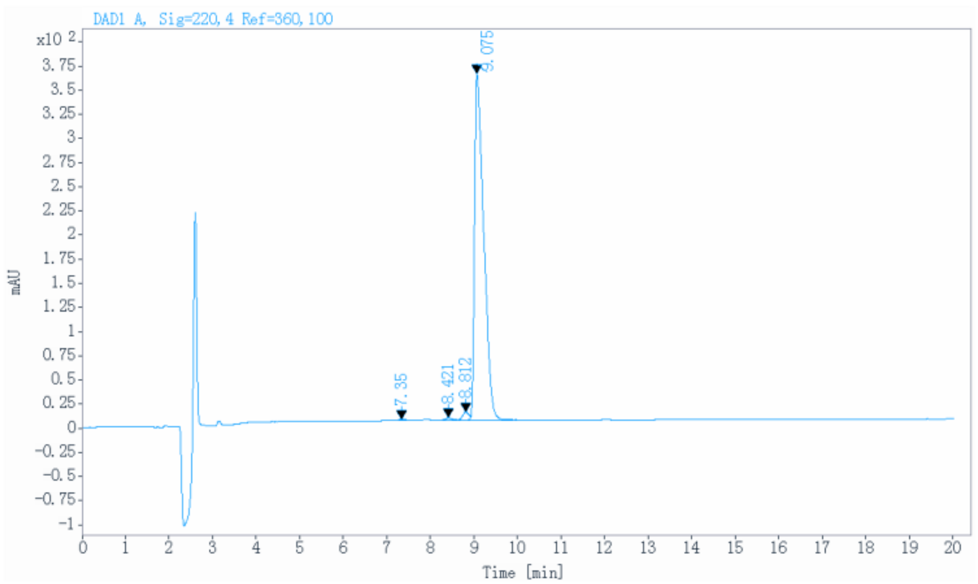

Signal: DAD1 A, Sig=220,4 Ref=360,100

RT[min]	Type	Width[min]	Area	Height	Area%	Name
7.350	FM	0.1718	16.6934	1.6197	0.3106	
8.421	VV E	0.1489	22.1798	2.1763	0.4126	
8.812	VV E	0.1535	78.7201	8.0911	1.4645	
9.075	VB R	0.2129	5257.6250	358.8497	97.8123	
		Sum	5375.2183			

Figure S3. MS spectroscopy and HPLC of DOTA-(2P-PEG$_4$)

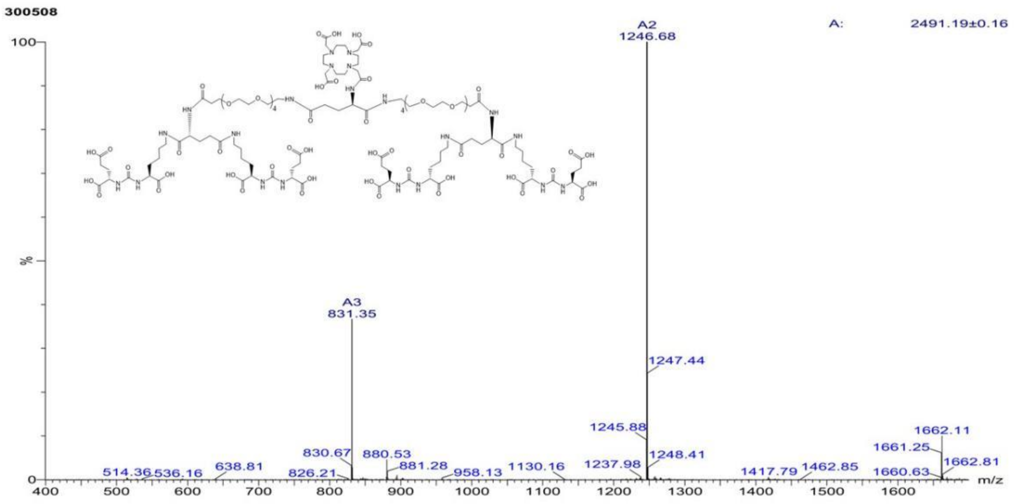

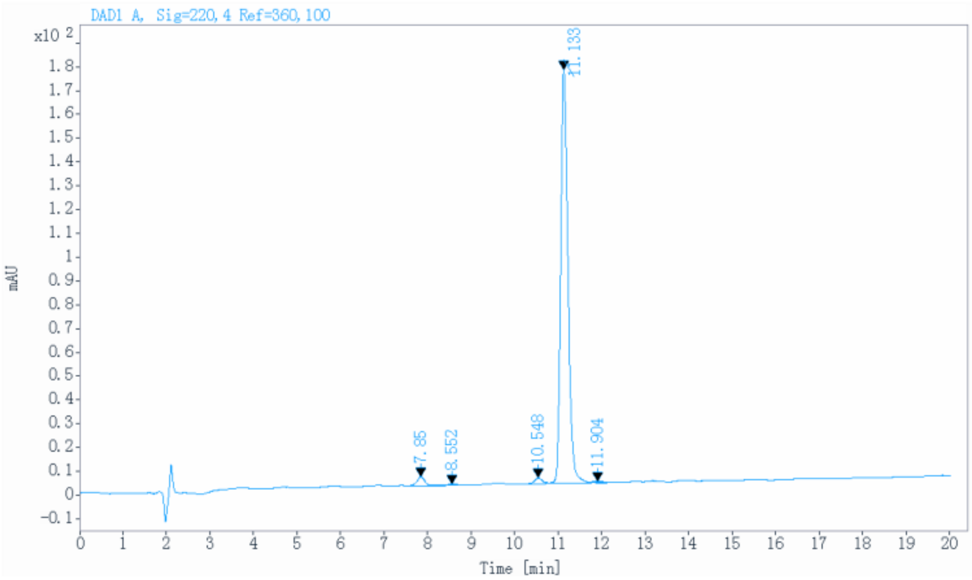

Signal: DAD1 A, Sig=220,4 Ref=360,100

RT[min]	Type	Width[min]	Area	Height	Area%	Name
7.850	BV R	0.1959	46.1268	3.7397	2.1827	
8.552	VB E	0.2763	10.4570	0.4994	0.4948	
10.548	BV E	0.2089	32.8172	2.3790	1.5529	
11.133	VV R	0.1751	2012.8818	173.7746	95.2491	
11.904	VB E	0.2580	10.9980	0.5215	0.5204	
		Sum	2113.2808			

Figure S4. MS spectroscopy and HPLC of DOTA-(2P-PEG4)2

Figure S1-4.DOTA- (1P-PEG$_4$), DOTA- (2P-PEG$_0$), DOTA- (2P-PEG$_4$) and DOTA-PEG4- (2P-PEG$_4$)$_2$ chemical structure, mass spectrometry analysis and chemical purity detection.

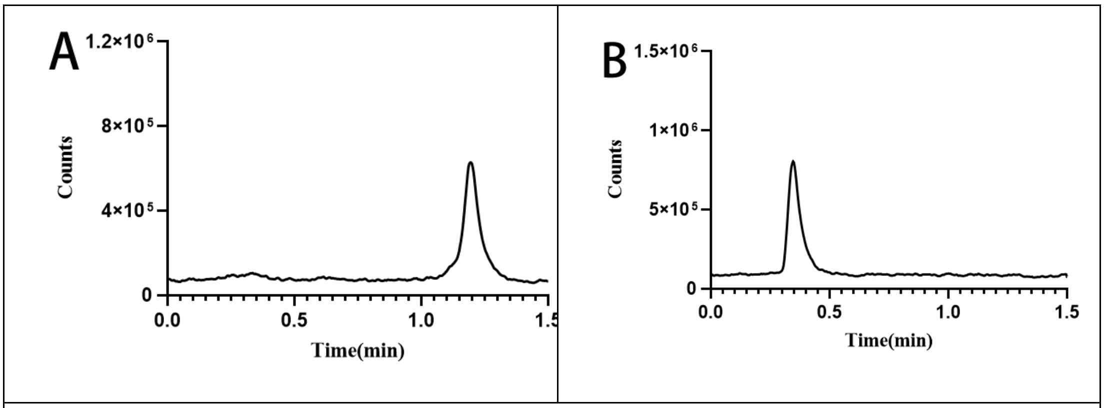

The radiochemical purity by thinlayer chromatography with the mobile phase of 0.1M tri-sodium-citrate to a PH of 5.0. (A):The TLC result of free ^{177}Lu. (B):The TLC result of [^{177}Lu]Lu-DOTA-(2P-PEG$_4$)$_2$.

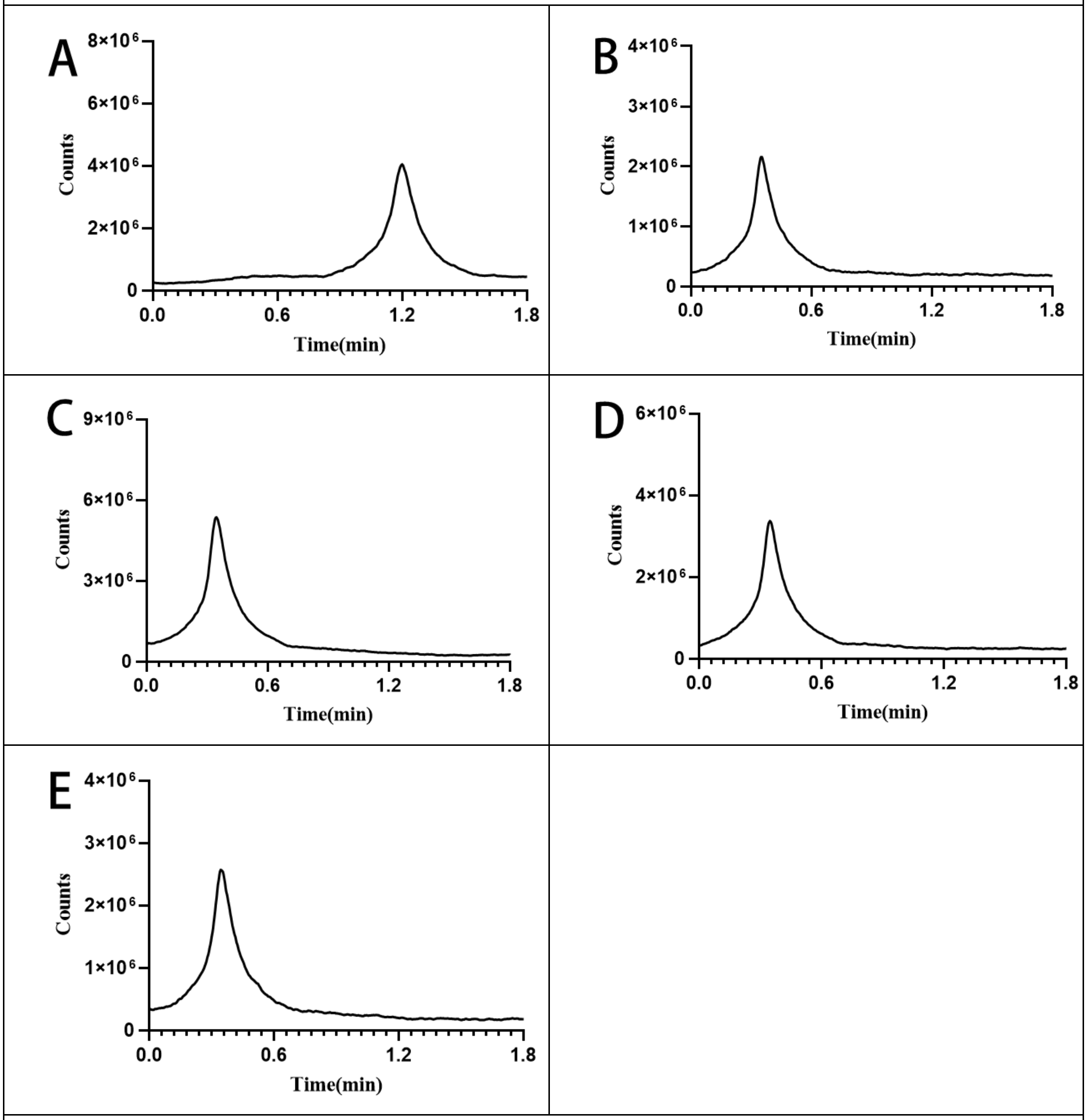

The radiochemical purity by thinlayer chromatography with the mobile phase of 0.1M tri-sodium-citrate to a PH of 5.0. (A):The TLC result of free ^{68}Ga. (B/C/D/E):The TLC result of [^{68}Ga]Ga-DOTA-(1P-PEG$_4$), [^{68}Ga]Ga-DOTA-(2P-PEG$_4$), [^{68}Ga]Ga-DOTA-(2P-PEG$_0$), [^{68}Ga]Ga-DOTA-(2P-PEG$_4$)$_2$

Figure S5. The radiochemical purity by thinlayer chromatography of free ^{68}Ga/^{177}Lu and

[^{177}Lu]Lu-DOTA-(2P-PEG4)2, [^{68}Ga]Ga-DOTA-(1P-PEG4), [^{68}Ga]Ga-DOTA-(2P-PEG0), [^{68}Ga]Ga-DOTA-(2P-PEG4) and [^{68}Ga]Ga-DOTA-(2P-PEG4)2

Table S1. [^{68}Ga]Ga-DOTA-(1P-PEG4), [^{68}Ga]Ga-DOTA-(2P-PEG0), [^{68}Ga]Ga-DOTA-(2P-PEG4), [^{68}Ga]Ga-DOTA-(2P-PEG4)2 corresponding tissue uptake values of PC-3 PIP tumor-bearing mice,expressed as Mean±SD, n=3.

	30min				60min				120min			
	DOTA-(1P-PEG4)	DOTA-(2P-PEG0)	DOTA-(2P-PEG4)	DOTA-(2P-PEG4)2	DOTA-(1P-PEG4)	DOTA-(2P-PEG0)	DOTA-(2P-PEG4)	DOTA-(2P-PEG4)2	DOTA-(1P-PEG4)	DOTA-(2P-PEG0)	DOTA-(2P-PEG4)	DOTA-(2P-PEG4)2
Heart	0.63±0.55	0.63±0.14	0.4±0.09	0.83±0.22	0.81±0.21	0.22±0.06	0.17±0.04	0.35±0.12	0.52±0.06	0.08±0.04	0.06±0.02	0.09±0.03
Liver	0.62±0.32	0.73±0.13	0.44±0.08	0.58±0.07	0.48±0.17	0.39±0.11	0.24±0.02	0.39±0.09	0.51±0.17	0.25±0.15	0.19±0.04	0.21±0.04
Spleen	1.17±0.84	2.76±0.13	3.34±0.72	2.24±0.21	1.18±0.39	0.85±0.42	0.91±0.38	0.97±0.33	1.46±1.49	0.26±0.12	0.59±0.15	0.23±0.09
Lung	1.26±0.64	1.98±0.34	1.21±0.31	1.75±0.13	1.78±0.63	0.55±0.09	0.46±0.06	0.83±0.16	0.86±0.6	0.19±0.03	0.27±0.07	0.24±0.07
Kidney	8.12±6.98	41.28±11.09	45.08±6.36	58.35±2.69	13.1±0.98	15.83±3.88	13.09±6.77	28.87±3.12	10.87±9.04	3.86±1.5	4.65±0.76	4.76±2.63
Stomach	0.66±0.42	0.78±0.19	0.5±0.09	0.77±0.1	0.76±0.06	0.33±0.06	0.2±0.1	0.49±0.26	0.69±0.07	0.17±0.12	0.16±0.07	0.11±0.02
Intestine	0.68±0.42	0.83±0.33	0.49±0.18	0.86±0.15	0.89±0.42	0.64±0.55	0.17±0.05	0.42±0.21	0.41±0.12	0.14±0.03	0.15±0.06	0.1±0.01
Muscle	0.34±0.25	0.91±0.6	0.46±0.31	0.48±0.24	1.48±1.56	0.53±0.58	0.18±0.1	1.1±0.6	0.28±0.03	0.06±0.05	0.09±0.08	0.08±0.04
Bone	0.27±0.24	1.82±2.6	0.28±0.07	0.63±0.12	0.8±0.56	0.39±0.36	0.16±0.13	0.5±0.09	0.48±0.33	0.04±0.06	0.13±0.06	0.32±0.18
Salivary	0.83±0.44	1.2±0.28	0.78±0.17	0.73±0.31	0.82±0.13	0.34±0.11	0.31±0.1	0.61±0.16	0.75±0.59	0.14±0.03	0.16±0.02	0.17±0.03
Brain	0.11±0.12	0.12±0.08	0.04±0.01	0.11±0.01	0.39±0.4	0.05±0.01	0.03±0.01	0.04±0.01	0.17±0.03	0.05±0.05	0.01±0	0.03±0.03
Blood	3.1±3.22	5.21±4.25	1.35±0.04	2.23±0.42	2.19±0.36	2.24±2.65	1.25±0.39	0.75±0.41	6.11±4.84	2.78±1.8	0.69±0.28	1.14±1.43
Tumor	11.8±6.39	23.91±2.67	18.57±3.21	23.05±2.66	14.63±2.24	15.12±1.69	16.13±1.41	28.89±1.82	14.04±0.87	14.29±4.62	16.04±1.86	21.85±3.53

Values are expressed as % injected activity per gram of organ tissue. Each value represents the mean ± standard deviation.

[^{68}Ga]Ga-DOTA-(1P-PEG$_4$)

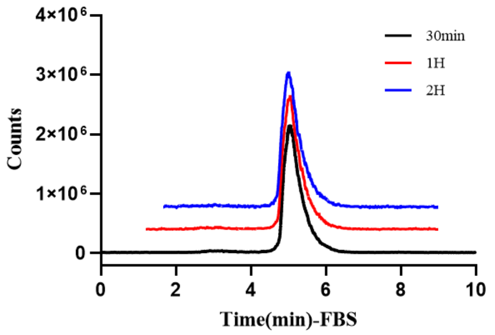

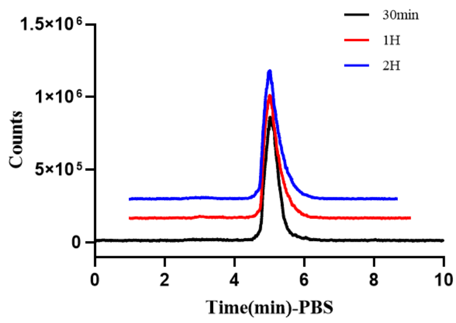

[^{68}Ga]Ga-DOTA-(2P-PEG$_0$)

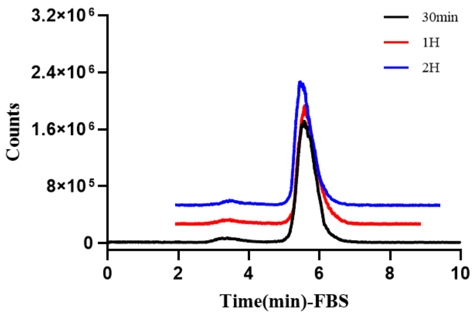

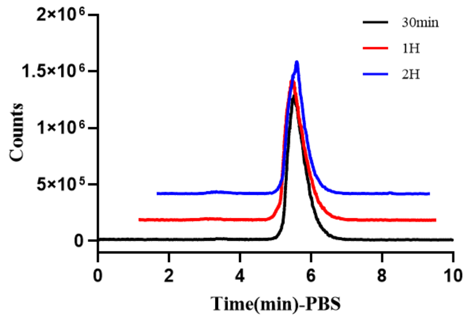

[^{68}Ga]Ga-DOTA-(2P-PEG$_4$)

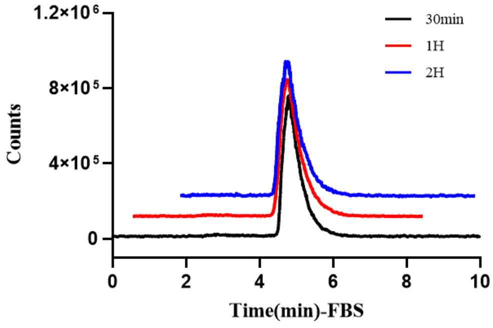

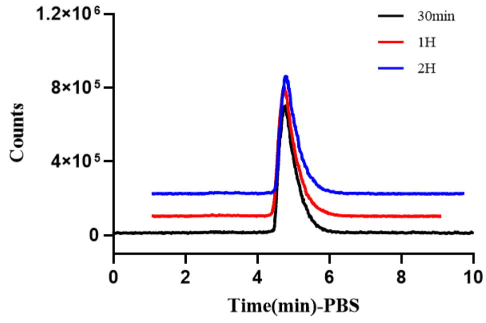

[^{68}Ga]Ga-DOTA-(2P-PEG$_4$)$_2$

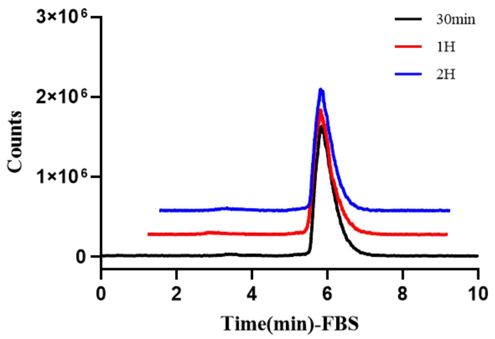

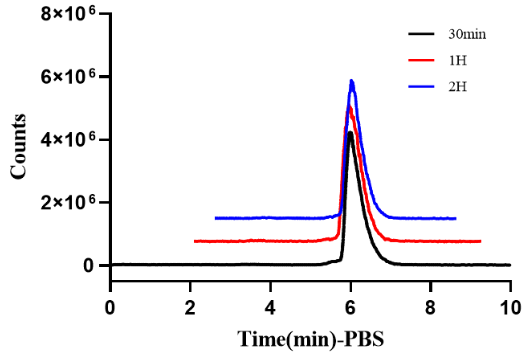

Radiochemical purity of [^{68}Ga]Ga-DOTA-(1P-PEG$_4$), [^{68}Ga]Ga-DOTA-(2P-PEG$_4$), [^{68}Ga]Ga-DOTA-(2P-PEG$_0$) and [^{68}Ga]Ga-DOTA-(2P-PEG$_4$)$_2$ incubated in PBS/FBS for 2 h at 37**°C**

Figure S6. The radiochemical purity by Radio-HPLC of [^{68}Ga]Ga-DOTA-(1P-PEG$_4$),

[^{68}Ga]Ga-DOTA-(2P-PEG$_0$), [^{68}Ga]Ga-DOTA-(2P-PEG$_4$) and [^{68}Ga]Ga-DOTA-(2P-PEG$_4$)$_2$.
